# PNPLA3 Single Nucleotide Polymorphism Prevalence and Association with Liver Disease in a Diverse Cohort of Persons Living with HIV

**DOI:** 10.3390/biology10030242

**Published:** 2021-03-21

**Authors:** Kenneth E. Sherman, Susan D. Rouster, Heidi Meeds, Javier Tamargo, Jun Chen, Richard Ehman, Marianna Baum

**Affiliations:** 1 University of Cincinnati College of Medicine, Cincinnati, OH 45267, USA; susan.rouster@uc.edu (S.D.R.); Heidi.Meeds@uc.edu (H.M.); 2 Robert Stempel School of Public Health, Florida International University, Miami, FL 33199, USA; jtama025@fiu.edu (J.T.); baumm@fiu.edu (M.B.); 3 Mayo Clinic, Department of Radiology, Rochester, MN 57058, USA; Chen.Jun@mayo.edu (J.C.); ehman.richard@mayo.edu (R.E.)

**Keywords:** fatty liver, steatosis, HIV, coinfection, fibrosis, gene polymorphism

## Abstract

**Simple Summary:**

In this pilot study, we determined the prevalence of single nucleotide polymorphisms (SNPs) in the PNPLA3 gene among persons living with HIV (PLWH). Overall, approximately 40% of the population carries at least one “G” allele that is associated with development of fatty liver and progressive liver disease. The highest rates were observed in those with Hispanic ethnicity. However, rates of steatosis (fatty liver) and liver fibrosis were relatively low when evaluated by magnetic resonance elastography with proton-density fat fraction measurement (MRE with PDFF). When putative NAFLD/NASH was present it was associated with the presence of the “G” allele.

**Abstract:**

In persons living with HIV (PLWH), there are multiple sources of liver injury. Gene polymorphisms of PNPLA3 (patatin-like phospholipase domain-containing protein 3) have been identified as an important cofactor for increased disease severity in both alcoholic and non-alcoholic steatohepatitis (NASH). We utilized a well-characterized cohort of ethnically and racially diverse patients with HIV to define the prevalence of PNPLA3 SNPs (single nucleotide polymorphism) (rs738409), and to determine the relationship to hepatic steatosis and liver fibrosis. Steatosis was determined using MRI-PDFF (magnetic resonance imaging-determined proton density fat fraction) and fibrosis was estimated using MR Elastography (MRE). From the Miami Area HIV Study (MASH) cohort, 100 HIV positive participants and 40 controls (HCV/HIV = 20; HCV and HIV negative = 20) were evaluated. Nearly 40% of all participants carried the variant G allele associated with increased liver disease severity and 5% were homozygotic GG. The variant SNP occurred most frequently in those self-identified as Hispanic compared to white or Black participants. Hepatic steatosis (>5% fat) was present significantly more often in those without HIV vs. those with (*p* < 0.001). Putative NAFLD/NASH was found to be present in 6% of tested subjects, who were HIV monoinfected. BMI was lower in those that carried the G allele for PNPLA3. This finding suggests that PNPLA3 may be an independent component to NAFLD (non-alcoholic fatty liver disease)/NASH development and longitudinal follow-up of the cohort is warranted.

## 1. Introduction

Steatohepatitis from alcoholic and non-alcoholic etiologies has emerged as a significant health problem in the United States and elsewhere. Steatohepatitis is defined by the presence of hepatic steatosis (>5% of hepatocytes) with evidence of liver injury, suggested by either serum transaminase elevation, or by histologic criteria. Since the etiology (alcoholic vs. non-alcoholic) steatohepatitis (ASH vs. NASH) can only be distinguished by history of alcohol use, there is often overlap in the diagnosis and disease outcomes. Recently, a gene polymorphism in the PNPLA3 (patatin-like phospholipase) coding domain (rs738409) has been identified as a key predictor of liver disease progression from fatty liver to steatohepatitis, in the setting of both alcoholic and non-alcoholic liver disease [1,2]. The gene, which is present on chromosome 22, is responsible for the production of a triacyl lipase that appears to play a central role in fat metabolism and insulin resistance. Putative mechanisms that have been suggested include alteration of gut barrier, reduction in choline availability, increases in short-chain fatty acids, and alterations in bile metabolism affecting farnesoid X receptor (FXR) signaling [3].

In persons living with HIV (PLWH), liver disease is a major cause of morbidity and mortality, even in the era of highly effective antiretroviral therapies [4]. The NA-ACCORD cohort reported mortality rates of between 6 and 11/100,000 person years for various subgroups of patients with viral coinfections [5]. Indeed, some antiviral medications may enhance risk of liver injury by a variety of mechanisms including weight gain with increased insulin resistance [6,7]. There are relatively limited data regarding the role of PNPLA3 single nucleotide polymorphisms (SNPs) in the development of steatosis, liver injury and fibrosis among those with HIV infection [8,9].

In a prior publication, we first described the presence of PNPLA3 SNPs in a cohort of HCV/HIV coinfected persons undergoing treatment for HCV. The aberrant allele (homozygotic or heterozygotic) was present in 34% of the study population. For that study, we developed, validated and utilized a unique method for determination of PNPLA3 SNPs using high resolution melting point (HRM) analysis. Unfortunately, the validation cohort did not provide information regarding measurement of hepatic steatosis, and data regarding alcohol use were lacking. Furthermore, assessment of liver injury was confounded by the presence of concomitant HCV infection in all study participants [9].

To further our investigation of the role and importance of PNPLA3 gene polymorphisms in PLWH, we employed a unique cohort based in South Florida, the MASH (Miami Studies on HIV) cohort. HIV infection rates and mortality are among the highest in the nation [10]. This cohort accessed detailed information regarding alcohol and drug use, and utilized magnetic resonance elastography (MRE) with magnetic resonance imaging-determined proton density fat fraction (MRI-PDFF) to evaluate the liver for both fibrosis and steatosis, respectively. In this manuscript, we focus on the baseline prevalence and associations of PNPLA3 with a subset of the cohort related to steatosis, hepatic injury, and fibrosis. These data will be used to power future longitudinal analyses.

## 2. Methods

### 2.1. Study Cohort

We evaluated participants enrolled in the MASH cohort, which comprises an ethnically and racially diverse population from South Florida which is primarily represented by Black and Hispanic minority groups. The cohort consists of more than 1000 enrollees who provided demographic and lifestyle information using a variety of well-validated survey instruments, as well as blood samples. Study participants underwent evaluation of liver fibrosis and liver fat as described below. For this analysis, we focused on evaluation of 100 randomly selected participants with HIV monoinfection plus a smaller group of controls with HCV/HIV coinfection or virus negative (neither HCV or HIV). Alcohol consumption was determined with AUDIT [11]. Scores greater than 8 were considered suggestive of an alcohol (vs. non-alcohol) etiology. Key cohort inclusion criteria allowed enrollment of male and female adult participants who were HBsAg negative. Study enrollment occurred between October 2016 through 2020. All enrolled subjects provided informed consent and the NIDA-funded cohort study is approved by the Florida International University Institutional Review Board.

### 2.2. PNPLA3 SNPs

The methodology for determination of PNPLA3 SNPs, and its validation have been previously described [9]. Briefly, PBMCs (peripheral blood mononuclear cells) from the study participants were isolated from peripheral blood collected in CPTs (cell preparation tubes) (Becton Dickinson Co., Franklin Lakes, NJ, USA), according to the manufacturer’s directions. Cells were stored at −80 °C until genomic DNA extraction using a Qiagen QIAamp DNA Blood Mini kit. A Bio-Rad CFX96 thermocycler was used for real-time PCR, amplifying region 43928824-43928869 located on chromosome 22. The amplicon products are subjected to heat shifts to produce a high-resolution melting curve. Using this method, three genotypes of rs738409 can be detected representing CC, CG, or GG SNPs. The CC SNP is wildtype and the GG SNP represents the variant allele of interest. CG is a heterozygotic blend.

### 2.3. Determination of Hepatic Steatosis and Fibrosis

Study subjects were evaluated for hepatic steatosis using MRI-PDFF and for fibrosis using MRE. Studies were performed on a Siemens 3T MAGNETOM Prisma device. Steatosis was defined as greater than 5% fat, and graded using standard histologic criteria (Stage 1; 5–33%; Stage 2; 34–65%; Stage 3; ≥66%). Hepatic fibrosis was determined by measurement of liver stiffness which is highly associated with fibrosis stage. Results of > 4.5 kPa were classified as cirrhosis and ≥3.7 kPa as advanced fibrosis [12]. The cutoff for any fibrosis (F1) was 2.9 kPa. Additionally, the NAFLD Fibrosis Score (NFS) was calculated for each enrolled participant. The score utilizes selected laboratory values (serum glucose, platelet count, albumin, AST/ALT ratio) and readily available patient characteristics (age, BMI, and diabetes status). It uses the formula:

−1.675 + 0.037 × age (years) + 0.094 × BMI (kg/m2) + 1.13 × IFG/diabetes (yes = 1, no = 0) + 0.99 × AST/ALT ratio − 0.013 × platelet count (×10^9^/L) − 0.66 × albumin (g/dL) [13]. The presence of diabetes was defined as fasting blood glucose > 126. Levels above 0.675 suggest presence of significant fibrosis.

### 2.4. Laboratory Assessments

Fasting blood samples were obtained from all participants and used to measure routine laboratory values by Quest Diagnostics Laboratory, including chemistry, metabolic panels, platelet and CD4 cell counts, and viral loads.

### 2.5. Insulin Resistance

Insulin resistance (IR) was calculated using the triglyceride to glucose (TyG) Index. The TyG Index was calculated by the formula ln [fasting triglycerides (mg/dL) × FPG (mg/dL)]/2. Levels less than or equal to 4.68 do not indicate presence of insulin resistance while higher levels suggest that IR is present [14].

### 2.6. Statistical Methods

Categorical and continuous data were measured and compared using appropriate non-parametric and parametric methods to include one-way analysis of variance (ANOVA) with comparison of means, and Chi-square and/or Fisher exact tests. Regression models were developed using both best subset regression methods (univariate) and linear models. A *p*-value of <0.05 using a two-tailed hypothesis was utilized to determine significance. *p*-values above this cutoff were designated as “not significant (n.s.)” A power calculation was performed to determine the number of subjects that would identify a 20% difference in the G allele SNP frequency when the allele was present in 40% of the population. A sample size of 100 yielded a power of 0.973 with an alpha = 0.05 to detect this difference. Analyses were performed using Statistix 10.0 software (Analytical Software, Tallahassee, FL, USA).

## 3. Results

### 3.1. Study Participants

The study population (n = 140) represented a subset of the MASH cohort as described above, targeting a sampling of those with HIV monoinfection (n = 100), with HCV/HIV coinfection (n = 20) and HCV/HIV negative controls (n = 20). Overall, the mean age was 52.2 years (range 23–69 years). The majority of participants were Black, non-Hispanic (66.4%) while 29% self-defined their ethnicity as Hispanic. White, non-Hispanic participants constituted 7.9% of the study population. These numbers are reflective of the MASH cohort as a whole and include nearly universal antiretroviral therapy, as reflected by the very low median HIV viral loads shown below. Table 1 shows key demographic and laboratory characteristics of the individual disease groupings. The groups were well-matched overall. Only AST levels were noted to be higher in the HCV/HIV coinfected group.

### 3.2. PNPLA3 Genotype Distibution

**Figure 1:**
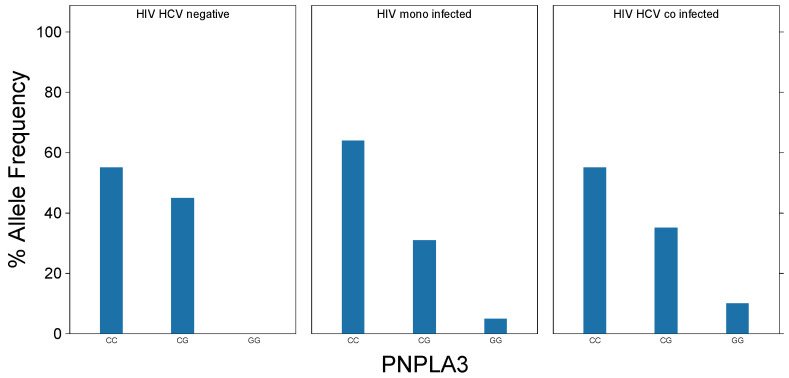
Gene frequency of PNPLA3 alleles by group.

The overall distribution of PNPLA3 SNPs was determined. The CC (wildtype) SNP was present in 86 participants (61.4%). The majority of the remainder (33.6%) were heterozygotic CG. Only seven subjects (5%) were homozygotic for the PNPLA3 GG SNP. Figure 1 demonstrates the distribution of PNPLA3 SNPs in the study population, including breakdown by disease group. The SNP gene frequency was not associated with the disease group (*p* = 0.49). The presence of non-wildtype SNPs (CG/GG) was highly associated with race/ethnicity. While Caucasian (White) participants were not statistically different to Blacks, those self-identified as Hispanic had a much higher gene frequency of non-wildtype alleles (Black, non-Hispanic vs. Hispanic, *p* < 0.05). Most participants with the homozygotic GG SNP for PNPLA3 (n = 5/7, 71.4%) were classified as Hispanic.

### 3.3. Steatosis, Fibrosis, and HIV

The presence of steatosis (>5% fat) was significantly more frequent among those without HIV infection vs. those with HIV (55% vs. 13.8%, respectively; *p* < 0.001). It was independent of the presence or absence of HCV coinfection (*p* = 0.48). Best subset regression models were utilized to determine which unforced variable factors were associated with the percent of liver fat. In best subset regression models that included PNPLA3 SNPs, HCV status, HIV status, alcohol use determined by AUDIT, age, sex at birth, insulin resistance, race/ethnicity, BMI, and diabetes (fasting blood glucose > 126), models that included PNPLA3 SNPs, HIV, ethnicity, diabetes, sex at birth, and BMI yielded the best overall model. In Table 2, a linear regression model was utilized to determine which of these factors was most important. Only BMI and HIV status were significant while other factors fell out of the model.

A subset of persons with steatosis includes those with NAFLD/NASH, defined herein as steatosis plus hepatic fibrosis without other known etiology. Using the same baseline factors, we utilized the linear model to predict those associated with the NAFLD Fibrosis Score. The NFS has been promulgated as a way to identify those with NAFLD/NASH. In this model, only BMI and fasting glucose were associated with the NFS (*p* < 0.05). The presence of PNPLA3 SNPs was not associated with increased NFS, either in univariate analysis or in this model. An alternative method to assess presence of NAFLD/NASH is to focus on subjects with steatosis determined by MRI-PDFF who also have hepatic fibrosis on MRE. Overall, 12.6% of the sampled cohort had fibrosis on MRE, most in the HCV/HIV coinfected group as shown in Table 1. However, only six participants in the HIV monoinfected group had both steatosis and fibrosis without HCV coinfection, suggesting that NAFLD/NASH is a relatively uncommon finding at this time. Two carried the PNPLA3 variant SNP, one as a heterozygotic and the other as a homozygotic allele. Neither participant had an AUDIT greater than 8, and neither was identified as NAFLD/NASH using the NFS score algorithm. Their BMI was significantly lower than (31.3 vs. 39.1) for those carrying the “G” allele.

## 4. Discussion

The primary focus of this pilot study was the determination of the gene frequency of polymorphisms in the PNPLA3 gene, which is implicated as an important factor in liver disease progression in patients with ASH and NAFLD/NASH. Using the baseline data of a longitudinal cohort, we sought to define the prevalence of the SNP in an ethnically diverse population infected with HIV. These data are critical to the design and follow-up of a longitudinal cohort that is being evaluated for development of hepatic steatosis and hepatic fibrosis. Overall, 40% of the tested participants carried the “G” allele, but significant higher rates were observed in those of Hispanic ethnicity compared to white and black participants. Saab et al. reviewed the literature regarding increased prevalence and severity of NASH in the Latino population [15]. The presence of the “G” allele was also associated with accumulation of fat in the liver, and was most prominent in those homozygotic for the “G” SNP [16]. In our survey cohort, 71.4% of the participants carrying the homozygotic “GG” alleles were Hispanic. Therefore, we would predict that a high proportion of our study population would have NAFLD/NASH.

Though there are a number of publications that attempt to define the prevalence and importance of fatty liver among PLWH, there are a confusing array of definitions and techniques that makes comparability and interpretation difficult [17]. The prevalence of fatty liver varies greatly by the methodology utilized. Many publications report data derived from ultrasound which is relatively insensitive to low levels of steatosis and confounded by other causes of echogenicity. The gold standard is liver biopsy, but this cannot reasonably be accomplished in large cohort studies. MRI-PDFF has emerged as the most sensitive and specific non-invasive measure of hepatic steatosis [18]. The literature regarding the frequency of NAFLD/NASH among PLWH is also highly variable in terms of frequency and importance, in part because definitions are not equal across studies. The Multicenter AIDS Cohort Study (MACS) failed to find any association of NAFLD/NASH with HIV viral detection or CD4 count. Fibrosis determined by transient elastography was present in 27% of participants with concomitant steatosis in a cohort study in China, but there was no relationship to CD4 count, the duration of HIV infection or the diagnosis of AIDS [19]. A Johns Hopkins HIV-infected cohort found that alcohol use and BMI were associated with hepatic steatosis and that this resolved with weight loss [20]. Among HIV-infected patients with abnormal ALT who underwent liver biopsy, 65% had steatosis but only 26% met histologic criteria for NASH [21]. Among 27 subjects who had fatty liver on ultrasound and subsequently underwent liver biopsy, 10 had no or minimal steatosis. NAFLD was associated with waist circumference and triglyceride levels but not with HIV parameters. Interestingly, the authors noted that NAFLD was much less common in African-Americans compared to White PLWH [22]. As our cohort was heavily weighted towards Black participants, this may have contributed to our observation that NAFLD/NASH prevalence was quite limited. Overall, these data from others suggest that steatosis is largely driven by BMI and/or alcohol use and that the presence of HIV does not significantly impact this outcome. Our data support this finding as well. However, this pilot study was designed to be hypothesis-generating in support of a larger longitudinal study that might better define the role of PNPLA3 and other SNPs on the development of liver disease in PLWH.

## 5. Conclusions

We hypothesized that PNPLA3 would be highly prevalent, and an important factor in development of NAFLD/NASH in our racially and ethnically diverse cohort. PNPLA3 variant SNPs were present in nearly 40% of the participants evaluated in this cohort. However, NAFLD/NASH appeared to be uncommon among those carrying the SNP. However, among the participants having putative NAFLD/NASH (as opposed to simple steatosis) the SNP was present at a lower BMI among PLWH, suggesting that it may represent an independent factor for disease progression to the more severe form of the disease process.

While the role of routine PNPLA3 testing is unclear based upon this analysis, further evaluation of larger datasets appears warranted. It is possible to speculate that persons with HIV, at risk by virtue of elevated BMI or diabetes, have increased risk of progressive hepatic fibrosis. This would need to be established in longitudinal cohort analysis, which is planned for the MASH cohort at a later date.

## Figures and Tables

**Table 1:** Demographics and key clinical and laboratory findings.

	HIV	HCV/HIV	HCV & HIV Negative	*p*-Value
	(n = 100)	(n = 20)	(n = 20)	
Age (years, median, range)	53 (23–69)	52 (27–65)	52 (40–62)	n.s
Sex (% male at birth)	63	60	35	n.s
Race/Ethnicity (%)				n.s.
Black, non-Hispanic	66	65	70	
White, Hispanic	18	20	20	
White, non-Hispanic	10	0	5	
Other	6	15	5	
ALT (Median, range)	18 (7–72)	25 (8–120)	19 (8–77)	n.s.
AST (Median, range)	21 (11–58)	30 (14–103)	26 (13–41)	0.03
BMI (Median)	28.1	30	31.6	n.s.
AUDIT (Median)	3.0	2.5	4.5	n.s.
CD4 (Median)	556	580	n/a	n.s.
HIV Viral Load (Median Copies/ml)	19	19	n/a	n.s.
TyG Index (median)	4.62	4.57	4.64	n.s.
Steatosis (>5% fat)	13.3%	20%	55%	0.001
Hepatic Fibrosis (%)	10.1	35.0	0	0.0016
PNPLA3 Genotype (%)				
CC	64	55	55	
CG	31	35	45	
GG	5	10	0	

**Table 2:** Regression model of factors associated with steatosis.

Characteristic	*p*-Value
Ethnicity/Race	0.88
BMI	0.0001
Diabetes	0.65
HIV	0.007
Sex (at birth)	0.85

## Data Availability

The data presented in this study are available on request from the corresponding author.
